# Ghrelin Receptors Enhance Fat Taste Responsiveness in Female Mice

**DOI:** 10.3390/nu13041045

**Published:** 2021-03-24

**Authors:** Ashley N. Calder, Tian Yu, Naima S. Dahir, Yuxiang Sun, Timothy A. Gilbertson

**Affiliations:** 1 Burnett School of Biomedical Sciences, College of Medicine, University of Central Florida, Orlando, FL 32827, USA; Ashley.Calder@ucf.edu (A.N.C.); Naima.Dahir@ucf.edu (N.S.D.); 2 Department of Cell & Developmental Biology, University of Colorado Anschutz Medical Campus, Aurora, CO 80045, USA; tian.yu@ucdenver.edu; 3 Department of Nutrition, Texas A&M University, College Station, TX 77843, USA; yuxiangs@tamu.edu; 4 Department of Internal Medicine, College of Medicine, University of Central Florida, Orlando, FL 32827, USA

**Keywords:** taste, ghrelin, diet, metabolism, fat

## Abstract

Ghrelin is a major appetite-stimulating neuropeptide found in circulation. While its role in increasing food intake is well known, its role in affecting taste perception, if any, remains unclear. In this study, we investigated the role of the growth hormone secretagogue receptor’s (GHS-R; a ghrelin receptor) activity in the peripheral taste system using feeding studies and conditioned taste aversion assays by comparing wild-type and GHS-R-knockout models. Using transgenic mice expressing enhanced green fluorescent protein (GFP), we demonstrated GHS-R expression in the taste system in relation phospholipase C ß2 isotype (PLCβ2; type II taste cell marker)- and glutamate decarboxylase type 67 (GAD67; type III taste cell marker)-expressing cells using immunohistochemistry. We observed high levels of co-localization between PLCβ2 and GHS-R within the taste system, while GHS-R rarely co-localized in GAD67-expressing cells. Additionally, following 6 weeks of 60% high-fat diet, female *Ghsr^−/−^* mice exhibited reduced responsiveness to linoleic acid (LA) compared to their wild-type (WT) counterparts, while no such differences were observed in male *Ghsr^−/−^* and WT mice. Overall, our results are consistent with the interpretation that ghrelin in the taste system is involved in the complex sensing and recognition of fat compounds. Ghrelin-GHS-R signaling may play a critical role in the recognition of fatty acids in female mice, and this differential regulation may contribute to their distinct ingestive behaviors.

## 1. Introduction

Ghrelin is a peptide hormone primarily produced by the endocrine cells in the stomach, with its most established function associated with the stimulation of food intake [1]. Circulating ghrelin levels rise between meals, which peak during a fasting state and fall within one hour after a meal [2]. Instead of directly reflecting the physiological fasting level, ghrelin is generally considered to be a meal anticipation signal, a food-entrainable circadian clock in both humans and mice [3,4]. Despite this fact, ghrelin’s actual role in both metabolic and feeding behaviors remains unclear. Interestingly, both fasted human and rodent models display elevated taste thresholds compared to their fed counterparts [5,6]. These studies are coincident with elevated ghrelin levels, suggesting a role for hormones such as ghrelin in impacting taste detection. However, this physiological connection between ghrelin and taste sensitivity, if any, is largely unexplored.

The current understanding of ghrelin’s orexigenic and metabolic effects is focused on its actions in the hypothalamus of the brain, which has been eloquently reviewed [7]. Interestingly, experimenters using an alternate *Ghsr* knockout model observed that the ghrelin receptor (growth hormone secretagogue receptor (GHS-R))-knockout *(Ghsr*^−/−^ KO) mice were resistant to high-fat diet (HFD)-induced obesity, with a reduction in food intake [8]. From these findings, one might predict that at least part of the diet-induced-obesity (DIO) resistance observed in these *Ghsr^−/−^* mice was due to a lower HFD intake [8]. In contrast, the *Ghsr^−/−^* mice by Sun et al. [9,10], which are used in the present study, showed no significant changes in food intake after being on a 35% high-fat diet for 10 weeks. To determine whether a 60% HFD elicits caloric intake or body weight differences in *Ghsr^−/−^* male and female mice, we performed a 6-week feeding study. Further, we investigated whether *Ghsr^−/−^* mice have an altered responsiveness to the chemical cues contained in dietary fat.

Ghrelin signaling elements have already been found in taste buds, the primary taste-sensing organelle in the peripheral sensory system. First, ghrelin can be produced by the salivary glands, with subsequent excretion of the hormone into saliva [11]. Second, both ghrelin and GHS-R have been found in type I, II, III, and IV taste cells [12,13]. Ghrelin signaling has been shown to alter sensitivities to certain tastants in the brief-access lickometer test. Ghrelin KO mice have reductions in both NaCl aversion and intralipid preference [12], and *Ghsr^−/−^* mice have reduced sensitivities to NaCl and citric acid [13]. While NaCl (salt) and citric acid (sour) sensitivities also contribute to the overall gustatory experience, the reduction in intralipid responsiveness in ghrelin KO mice suggests that the ghrelin/GHS-R axis plays a role in the initial events surrounding the taste of fat.

Palatable foods rich in lipids are known to be attractive to humans and rodents. Lipids can be easily hydrolyzed to free fatty acids (FFAs) by lingual lipase provided by von Ebner’s gland in the oral cavity [14,15]. Additionally, there is a sufficient concentration of free fatty acids present in fat-containing food where they act as gustatory cues for dietary fat [16,17,18]. Over the past 20 years, molecular mechanisms of FFA recognition in the taste system have slowly emerged, with delayed rectifying potassium channels (DRKs), fatty-acid-sensitive G protein-coupled receptors (i.e., GPR40 and GPR120), and the fatty acid transporter CD36 as the top candidates for sensors of FFAs in the oral cavity [18,19,20,21,22]. The somatosensory system also contributes to the sensory detection of FFAs. Several FFAs of varying chain lengths have been reported to be able to induce calcium responses in trigeminal neurons [23]. Therefore, the idea that fat sensing occurs during the initial events in peripheral chemosensory pathways, playing a significant role in the overall flavor experience in foods, is gaining increasing popularity. However, whether fat taste sensing can be modulated by other factors, especially those related to the modulation of food intake, remains unclear. Given that ghrelin KO mice previously showed a decrease in lipid taste responsiveness [12] and considering the observed reduction of HFD intake in *Ghsr^−/−^* mice, we hypothesize that loss of ghrelin receptors in mice leads to a reduction in the peripheral signals carrying fat taste information emanating from the oral cavity. To test this, we examined whether GHS-R plays a role in taste-mediated fat detection by comparing taste responsiveness to linoleic acid (LA, the prototypical fatty acid stimulus and one that is abundant in food) in *Ghsr^−/−^* animals and their WT counterparts using conditioned taste aversion (CTA) assays.

## 2. Materials and Methods

### 2.1. Animals and High-Fat-Diet Feeding

Eight-week *Ghsr^−/−^* and littermate wild-type (WT) control mice were obtained from the laboratory of Dr. Yuxiang Sun, where the mice were backcrossed with a C57BL/6J background over 10 generations [10]. All mice were bred at the Laboratory Animal Research Center (LARC), and all procedures were approved by the Institutional Animal Care and Use Committees (IACUC) of Utah State University and the University of Central Florida. Our goal was to assess the effects of loss of GHS-R in mice that have been maintained on a high-fat diet. Therefore, *Ghsr^−/−^* and WT mice were fed a high-fat diet (HFD; 60% calories from fat, Research Diets D12492) for 6 weeks, with ad libitum access to food and water. Body weights were recorded at the beginning of the feeding study and then weekly until the end of the study. Body composition data were collected prior to the start of HFD feeding and immediately following completion of the feeding study using a Bruker minispec LF-50 body composition analyzer (Billerica, MA, USA). All mice were switched to a chow diet (Teklad rodent diet #8604) for a minimum of 2 days to facilitate the formation of a conditioned taste aversion to LA. A total of 37 WT mice (21 females, 16 males) and 29 *Ghsr^−/−^* mice (12 females, 18 males) were used.

### 2.2. Immunohistochemistry

To determine the expression pattern of GHS-R in cell types within the taste bud, adult PLCβ2-GFP and GAD67-GFP transgenic mice on a C57Bl/6 background were used. The PLCβ2-GFP mice were a generous gift from Dr. Nirupa Chaudhari (University of Miami School of Medicine), and the GAD67-GFP mice were purchased from the Jackson Laboratory (Bar Harbor ME). The PLCβ2-GFP and GAD67-GFP transgenic mice were deeply anesthetized with isoflurane and perfused transcardially with 4% paraformaldehyde in phosphate buffer (PB, pH 7.4). The tongues were excised and immersed in the same fixative for 1 h at room temperature first and cryo-protected in 30% sucrose in phosphate-buffered saline (PBS, pH 7.4) overnight. After cryoprotection, tissue sections containing circumvallate and fungiform papillae were embedded in OCT, frozen and sectioned at 20 µm using a cryostat, and mounted on Superfrost Microscope Slides (Fisher Scientific). After 3× 10 min rinses with PBS, the sections were blocked with 10% normal goat serum and 2% bovine serum albumin in PBST (PBS-0.05% Tween^®^ 20) for 1 h and incubated with 1:500 rabbit GHS-R (extracellular) (Alomone, Jerusalem, Israel) overnight in a blocking solution without Tween^®^ 20. Following another 3× 10 min rinsing with PBS, the sections were incubated with 1:500 goat-anti rabbit Alexa Fluor 594 (Invitrogen) for 2 h in the same diluent as the primary antibody. To validate the specificity of our antibody, *Ghsr^−/−^* mice served as controls for the immunofluorescence assays and treated in a similar fashion as the experimental sections. Subsequently, all the sections were rinsed 3× for 10 min each in PBS, counterstained with 1:2000 Hoechst 33342 (Invitrogen, A10027) in PBS for 10 min for nuclei staining, and mounted with Fluoromount G (Southern Biotech). We used a laser scanning confocal microscope (Zeiss, LSM710) equipped with 405, 488, 561, and 633 laser lines for images acquisition. Images were processed by ImageJ, and PLCβ2- and GAD67-positive taste cells were counted using the Cell Counter plug-in in ImageJ (V1.51s).

### 2.3. Conditioned Taste Aversion (CTA) Assay

**Figure 1:**
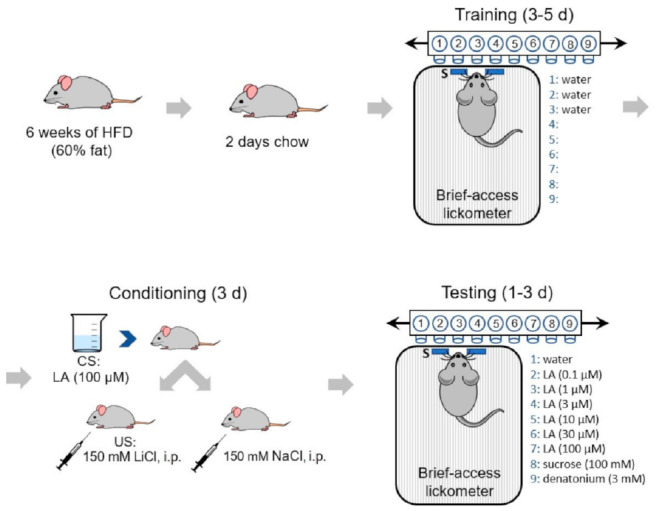
Conditioned taste aversion paradigm. Following 6 weeks of high-fat diet (HFD) (60%), the mice underwent the following conditioning paradigm. The mice were trained to lick from a Davis rig for 3–5 days prior to conditioning using water until they consistently licked the bottle during a 5 s interval. On conditioning days, mice were given free access to the unconditioned stimulus (100 µM linoleic acid (LA)) prior to an intraperitoneal injection of either 150 mM NaCl or LiCl (conditioned stimulus; 127 mg/kg). Mice in the LiCl treatment group were observed post-injection for signs of gastric distress. During testing days, mice were given access to LA at concentrations of 0.1, 1, 3, 10, 30, and 100 µM; 100 mM sucrose; 3 mM denatonium benzoate; and water in a randomized sequence. Mice had access to test solutions for 5 s followed by a rinse solution (water) for 2 s before presentation of the next test solution.

The scheme of our CTA assay is shown in Figure 1. Four groups of mice (*Ghsr^−/−^* females and males, WT females and males) were used in the study. Each group was further divided into two sub-groups to receive either LiCl (experimental manipulation, CTA) or NaCl (control) injections with the following sample sizes that successfully completed training: *Ghsr^−/−^* female LiCl (*n* = 7), NaCl (*n* = 4); *Ghsr^−/−^* male LiCl (*n* = 10), NaCl (*n* = 8); WT female LiCl (*n* = 9), NaCl (*n* = 6); and WT male LiCl (*n* = 8), NaCl (*n* = 7). The details of using CTA assays to assess the taste sensitivity were described previously [24]. Briefly, the whole paradigm consisted of three stages: training, conditioning, and testing. Mice had ad libitum access to water until 24 h prior to the first training day, when mice were started on a 23.5 h water restriction schedule for the whole duration of the experiment. On water-restricted days, 2 h after the start of training/conditioning/testing, animals were given 30 min access to water to facilitate rehydration. Training days were designed to familiarize mice to the lickometer chamber and testing procedures using water as the stimulus for the training trials (MS-160 Davis Rig gustatory behavioral apparatus, DiLog Instruments, Tallahassee, FL). Training was followed by three conditioning days, where animals were trained to avoid the conditioned stimulus (100 µM LA). Briefly, on each conditioning day, mice were first given 5 min access to 100 µM LA. Once the mice stopped licking, they were given the same solution orally with syringes. Immediately after the intraoral application, either 150 mM LiCl or 150 mM NaCl (control) was administered through intraperitoneal injections (20 mL/kg body weight). All mice receiving LiCl injections showed behavioral signs of gastric malaise within 10 min of the injection. There were three testing sessions (days 0, 1, and 2) performed. Day 1 data were reported, when mice were behaving more consistent after day 0, where significant neophobia was evident across all stimulus classes, but the associated aversion had not weakened. On the testing days, 9 bottles (8 stimuli and 1 water) were used on a Davis rig. To reduce olfactory cues, a fan was placed near the chamber to provide constant airflow and to serve as white noise. The effectiveness of the fan was evident as mice rarely accessed the spout without initiating licking behavior. The test session included 2 blocks of 9 trials (8 stimuli plus 1 water) with stimulus durations of 5 s, a water rinse of 2 s, and wait times for the first lick of 150 s. The stimulus order within each block was randomly assigned. Total numbers of licks per stimulus were summarized across the two trials per test session and normalized using a lick ratio (licks per test stimulus/licks to water) in order to account for individual variances in the water-restricted motivation across the mice. Zero-lick trials, while rare, were not included in subsequent analyses. Thus, all mice included in the data analysis sampled each stimulus at least once during each daily test session.

### 2.4. Stimuli

All taste stimuli were prepared from reagent-grade chemicals and presented at room temperature. In addition to water, there were 8 test stimuli in the study, which consisted of 0.1, 1, 3, 10, 30, and 100 µM LA; 100 mM sucrose; and 3 mM denatonium benzoate. All LA solutions were made fresh daily on conditioning/testing days. Sucrose and denatonium benzoate were made fresh on day 0 of testing.

### 2.5. Statistics

The normalized lick rates of female and male WT or *Ghsr^−/−^* mice were examined using two-way ANOVA treating the unconditioned stimulus (LiCl or NaCl) and days (day 1 or day 2) as between-subject variables. Test solutions (6 concentrations of LA) were treated as within-subject variables. The simple effects within test solutions were corrected with Bonferroni’s multiple-comparison test. Results are presented as the mean ± SEM. For body weights and MRI body composition analyses, the two-way ANOVA method with Bonferroni’s multiple-comparison test was used for correcting multiple comparisons. Unpaired t-tests were used in food intake analysis. The alpha value was set as 0.05. All the analyses were done using GraphPad Prism 7.

## 3. Results

### 3.1. GHS-R Is Expressed Predominantly in Type II Taste Cells

**Figure 2:**
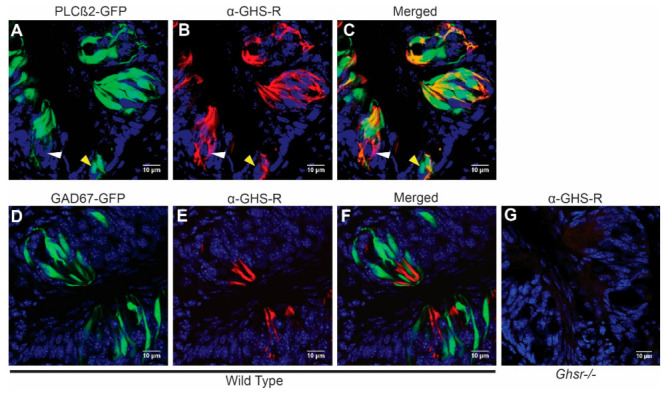
Growth hormone secretagogue receptor (GHS-R) is expressed in type II but rarely in type III taste cells of circumvallate papillae. (**A**–**C**) PLCβ2-GFP, green; anti-GHS-R, red; and merged images, respectively. In (**C**), the yellow arrow points to a representative taste cell that expresses PLCβ2 but not GHS-R, and the white arrow highlights a PLCβ2-negative, GHS-R-positive taste cell. (**D**–**F**) GAD67-GFP, green; anti-GHS-R, red; and merged images, respectively. (**G**) Anti-GHS-R antibody incubated on a representative section of circumvallate papillae from a GHS-R-deficient mouse (negative control). Nuclear staining is shown in blue in all figures.

**Figure 3:**
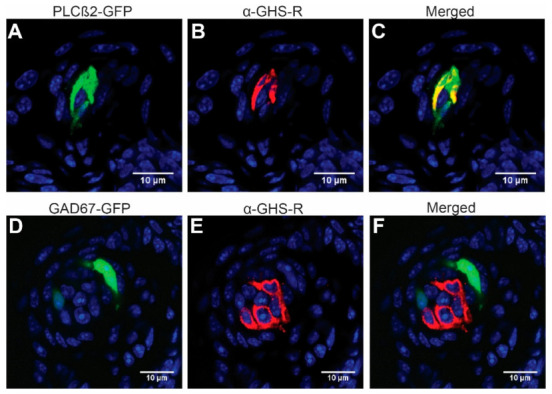
GHS-R is expressed in type II but rarely in type III taste cells of the fungiform papillae. (**A**–**C**) PLCβ2-GFP, green; anti-GHS-R, red; and merged images, respectively. (**D**–**F**) GAD67-GFP, green; anti-GHS-R, red; and merged images, respectively.

Although it was previously reported that the GHS-R antibody co-labels with markers from all taste cell types [12,25], here, we examined cell-type-specific extracellular GHS-R expression, again using PLCβ2-GFP and GAD67-GFP mice, which faithfully label type II and type III cells. As shown in Figure 2A–C, GHS-R was expressed in some but not all PLCβ2-positive type II cells from circumvallate papilla. In contrast, it was almost completely absent in GAD67-positive type III cells from circumvallate papilla (Figure 2D–F, Table 1). Immunohistochemistry from fungiform papillae showed a similar pattern (Figure 3). After counting GHS-R and PLCβ2 or GHS-R and GAD67 co-expression cells, we found that in circumvallate papilla, 71.1% of GHS-R cells were type II and 2.9% were type III, while in fungiform papilla, 100% of GHS-R cells that we counted were type II and 4.2% were type III (Table 1). This indicates that GHS-R is expressed mainly in type II and possibly in type I or other supportive basal cells but rarely in type III cells. We compared the GHS-R expression pattern in both sexes of mice; no obvious differences were observed.

### 3.2. Ghsr^−/−^ Males and Females Express Divergent Metabolic Phenotypes

**Figure 4:**
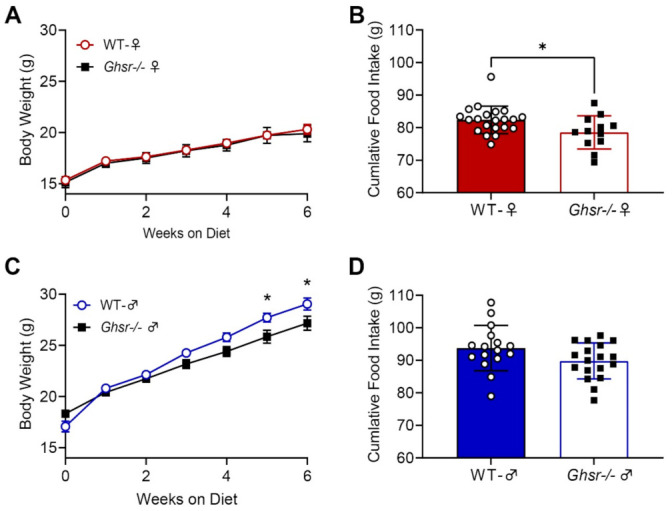
Body weight and food intake in wild-type (WT) and GHS-R-deficient mice on a high-fat diet. While not showing a significant difference in body weight (**A**), *Ghsr^−/−^* females consumed less compared to WT females (**B**). Alternatively, male *Ghsr^−/−^* mice (**C**,**D**) showed a decrease in body weight by week 5 of the HFD and no significant differences in food intake compared to their WT counterparts.* *p*-value < 0.05.

**Figure 5:**
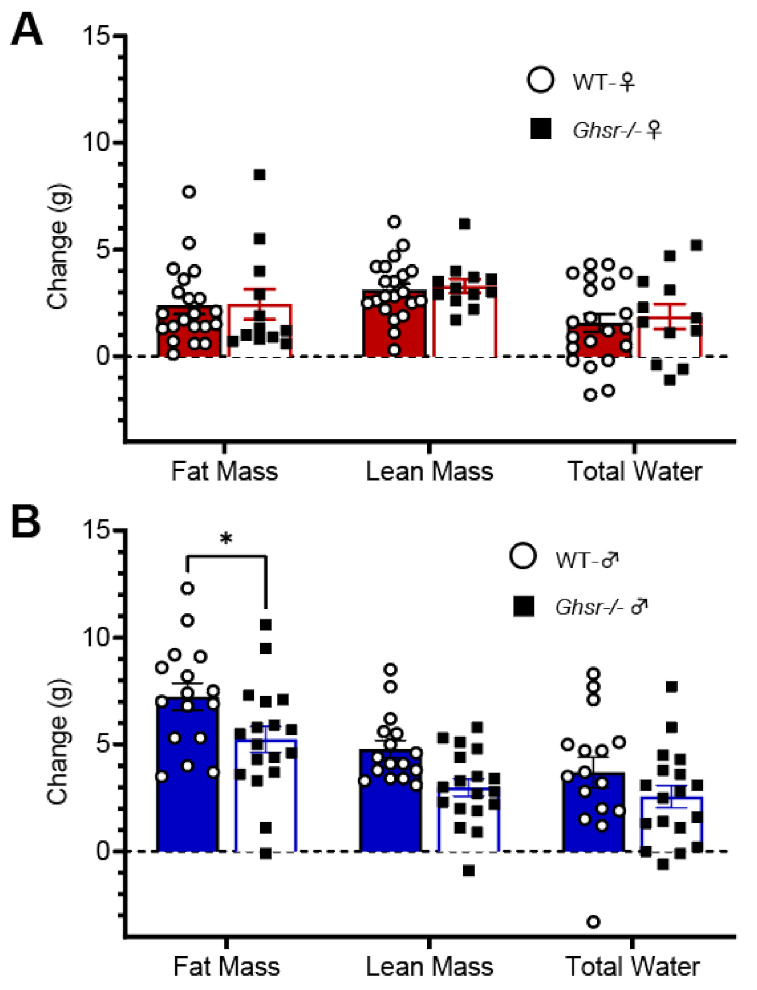
Body composition changes in WT and *Ghsr^−/−^* mice on a high-fat diet. Changes in body compositions calculated from data collected before the HFD and after 6 weeks of the HFD. (**A**). No significant changes in body composition were found in WT and *Ghsr^−/−^* females on 6 weeks of the HFD. (**B**). WT males gained more fat mass on 6 weeks of HFD compared to *Ghsr^−/−^* males.* *p*-value < 0.05.

*Ghsr^−/−^* and WT males and females were placed on 6 weeks of 60% high-fat diet (HFD) feeding. Female mice showed no significant differences in weight gain (F (1, 217) = 0.5382, *p* > 0.05) (Figure 4A). In contrast, however, *Ghsr^−/−^* males gained less weight on the HFD than WT males (F (1, 224) = 11.15, *p* < 0.01) (Figure 4C). While they did not gain weight, *Ghsr^−/−^* females consumed less HFD than their WT counterparts (WT 82.4 ± 0.9 g vs. *Ghsr^−/−^* 78.6 ± 1.5 g, *p* < 0.05) (Figure 4B). No significant changes in food consumption were observed between WT and *Ghsr^−/−^* males (WT 93.8 ± 1.7 g vs. *Ghsr^−/−^* 89.8 ± 1.3 g, *p* > 0.05) (Figure 4D). Studies have seen a similar metabolic phenotype for these *Ghsr^−/−^* males where they show reduced body weight but similar HFD consumption [9] These metabolic trends were further observed in the MRI body composition data where no significant changes were found between WT and *Ghsr^−/−^* females in fat, lean, or water mass (F (1, 93) = 0.2414, *p*> 0.05), (Figure 5A). *Ghsr^−/−^* males, however, showed a significant decrease in fat mass but not in water or lean mass (F (1, 96) = 13.14, *p* < *0*.001) (WT 4.8 ± 0.4 vs. *Ghsr^−/−^* 3.0 ± 0.4 *p* < 0.01; Figure 5B).

### 3.3. Female Ghsr^−/−^ Mice Show Reduced Avoidance to Linoleic Acid in CTA Assays

Since *Ghsr^−/−^ mice* are known to have altered feeding behavior and metabolic status, we hypothesized that the taste detection of fat contributes, at least in part, to this phenomenon by altering fatty acid responsiveness at the peripheral level. Therefore, we performed brief-access behavioral assays after forming a CTA to LA (conditioned stimulus, 100 µM LA) to investigate the alteration of taste responsiveness to LA in both sexes of *Ghsr^−/−^* and WT mice.

**Figure 6:**
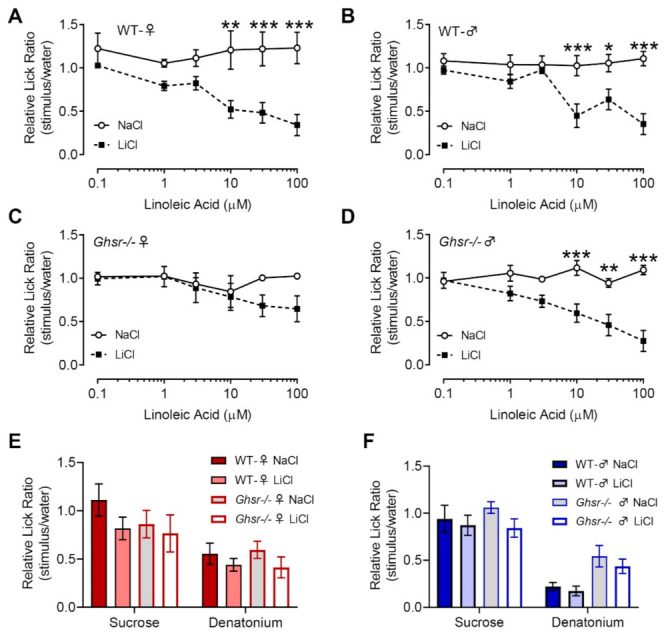
Linoleic acid responsiveness assessed in a conditioned taste aversion assay showed changes in *Ghsr^−/−^* female mice. (**A**). WT female mice *(n* = 15) revealed a significant aversion to LA at 10 µM, similar to that seen in WT males (*n* = 15) (**B**). *Ghsr^−/−^* females (*n* = 11) showed no significant differences in the LiCl compared to the NaCl group across all concentrations of LA (**C**). Male mice lacking GHS-R (*n* = 18) showed aversion at 10 µM LA, similar to WT mice (**D**). WT and *Ghsr^−/−^* females (**E**) and males (**F**) exhibited similar lick ratios to the control solutions, sucrose (100 mM), and denatonium benzoate (3 mM). * *p*-value < 0.05, ** *p*-value ≤ 0.01, and *** *p*-value < 0.0001.

Using the CTA assay with 100 µM LA as the conditioned stimulus, the WT female mice developed an aversion to LA at concentrations as low as 10 µM (F (1, 78) = 51.71, *p* < 0.0001) (Figure 6A). In contrast, *Ghsr^−/−^* female mice did not develop a significant aversion to LA (F (1, 54) = 3.085, *p* > 0.05) (Figure 6C), though there was evidence of an aversive profile at higher concentrations. These findings suggested that the LA taste responsiveness in female *Ghsr^−/−^* mice was reduced compared to the WT controls. Due to our immunohistochemical (IHC) findings showing high levels of co-localization between GHS-R and PLCβ2 (type II cells), we used two other G-protein-mediated tastants requiring PLCβ2, bitter and sweet, to test the overgeneralization of LA aversion to other tastants. The preference for the sweet stimulus sucrose and the rejection of the bitter stimulus denatonium benzoate showed no differences between the *Ghsr^−/−^* and WT animals (WT females (F (1, 28) = 3.097, *p* > 0.05); *Ghsr^−/−^* females (F (1, 18) = 0.7361, *p* > 0.05)) (Figure 6E).

Interestingly, the male *Ghsr^−/−^* mice did not display the reduced aversion to LA, as shown in the female *Ghsr^−/−^* mice, which corresponds with similar high-fat diet intake among the *Ghsr^−/−^* and WT males. As shown in Figure 6B, WT mice developed a normal taste aversion to LA, starting from 10 µM, and male *Ghsr^−/−^* mice presented a similar trend in LA aversion. As shown in Figure 6D, male *Ghsr^−/−^* mice showed evidence of aversion to LA, beginning at concentrations of 10 µM (WT males (F (1, 78) = 38.12, *p* < 0.0001); *Ghsr^−/−^* males (F (1, 96) = 55.72, *p* < 0.0001). These data suggest that reduction in LA taste responsiveness in *Ghsr^−/−^* mice is restricted to female mice, as in the case of females, loss of GHS-R did not affect behavioral responses to either sucrose or denatonium in the CTA assay (WT males (F (1, 26) = 0.5446, *p* > 0.05; *Ghsr^−/−^* males (F (1, 32) = 3.247, *p* > 0.05) (Figure 6F).

## 4. Discussion

It is well known that numerous hormones regulate eating behaviors through higher level processing in the brain. However, many of these same hormones, like ghrelin, are present in the circulatory system and have secondary targets throughout the peripheral systems involved in metabolism and food intake. A recent study has shown that neuronal specific deletion of GHS-R alone is able to prevent HFD-induced obesity in male mice [26]. Additionally, ghrelin has been shown to interfere with eating behavior at many levels. Bitter taste receptors and α-gustducin stimulate ghrelin secretion in the stomach, promoting consumption and then later delaying stomach emptying [27]. Centrally administered ghrelin (intracerebroventricular or intra-ventral tegmental area) acutely (3–6 h) increases chow and lard intake but not sucrose intake [28]. On the other hand, peripheral ghrelin injections (intraperitoneal) increase saccharin ingestion for 4 h post-injection [29]. While research has focused mainly on the role of ghrelin in macronutrient and caloric intake, less research has been done to understand whether the contributing role of ghrelin in orexigenic behaviors is due to manipulation of nutrient detection in the taste system. To better understand its role in taste (more specifically fat taste detection) and to limit off-target effects of ghrelin, we used a global *Ghsr^−/−^* mouse model to focus specifically on the ghrelin–GHS-R pathway.

In this report, we examined the effects of the ghrelin receptor, GHS-R, on the taste system. We showed that GHS-R is expressed in PLCβ2-positive type II taste cells but rarely in GAD67-positive type III taste cells, indicating possible interactions with sweet, bitter, umami, and fatty acid taste sensing. In addition, previous data by Sun et al. demonstrated that on a 35% HFD, *Ghsr^−/−^* males had caloric intake and body weight similar to WT counterparts [9]. To better understand metabolic changes in *Ghsr^−/−^* males and to further understand whether there are sex-dependent differences in these *Ghsr^−/−^* mice, we performed feeding studies and body composition measurements on both male and female *Ghsr^−/−^* and WT mice. Behaviorally, we observed differing roles of ghrelin among the sexes in *Ghsr^−/−^* mice. Following 6 weeks of a 60% HFD, *Ghsr^−/−^* males had significantly less fat mass compared to their WT counterparts, with no change in HFD intake. Additionally, *Ghsr^−/−^* female mice consumed less food than their WT counterparts, with no significant differences in weight gain or fat mass. Sex-dependent differences were also present in conditioned taste aversion assays, where *Ghsr^−/−^* females showed reduced aversion to LA but *Ghsr^−/−^* males showed no significant changes compared to WT mice.

Previous data published by Shin et al. reported the expression of ghrelin and GHS-R in all taste cell types using double-labeling of the GHS-R antibody and other taste cell-type-specific antibodies [13]. Our data support their finding that GHS-R co-localizes in PLCβ2 (type II)-expressing cells. Additionally, our data show GHS-R expressed in a subset of cells that did not express PLCβ2 (about 30% of GHS-R-expressing cells) and had little co-localization with GAD67 (type III), supporting their findings of GHS-R in non-PLCβ2-expressing cells such as type I and basal taste cells. Contrary to their findings, we observed little expression of GHS-R in type III cells. These differences, however, could be due to the use of different type III markers (neural cell adhesion molecule (NCAM) vs. GAD67) or a different methodology, as our study used a genetically expressed GFP under the control of a type III-specific gene (GAD67), and the previous study used dual-labeling of a type III marker and GHS-R. Our data provide new insight into the potential role of GHS-R in taste signaling. Relatively high levels of co-expression of GHS-R and PLCβ2 suggest a more targeted role of ghrelin/GHS-R in the taste system, as type II cells respond to G-protein-mediated tastes: bitter, sweet, umami, and fat.

CD36 and GPR120 are thought to be the primary receptors for the long-chain polyunsaturated fat taste pathway. The majority of ghrelin-expressing cells of the stomach express GPR120, and both GPR120 and long-chain unsaturated fatty acids have been shown to inhibit ghrelin secretion [30,31,32]. In addition, ghrelin-deficient mice exhibit decreased *Gpr120* expression in isolated taste buds [12]. These data together suggest a necessary crosstalk between ghrelin and fatty acid pathways to maintain metabolic balances. While it has been shown that fatty acid activation of GPR120 inhibits secretion of ghrelin, it may be that ghrelin also plays a role in sensing pathways for fatty acids in the oral cavity to help further drive metabolic needs. Future studies are needed to determine how or whether ghrelin/GHSR pathways interact with GPR120 to regulate both ghrelin secretion and fat taste sensitivity.

Cai et al. reported that ghrelin (*Ghrl^−/−^*)- and ghrelin O-acyltransferase-knockout (*Goat^−/−^*) male mice had reduced intralipid (a fat emulsion) sensitivity but did not appear to have altered preference for intralipid compared to their WT counterparts [12]. Additionally, they showed that ghrelin-deficient mice had reduced expression of fatty acid receptors (CD36 and GPR120) thought to play a crucial role in fat taste transduction, while they found no significant expression changes in the components of bitter, sweet, and umami taste pathways. Following this and other studies suggesting that *Ghsr^−/−^* mice are resistant to high-fat-diet-induced obesity [8], we focused on the role of GHS-R in lipid sensing using *Ghsr^−/−^* mice. Lipids can be easily hydrolyzed to FFAs by lingual lipase, and FFAs exist in food at concentrations that can be detected by taste cells. For rodents, fatty acid solutions by themselves are less preferred [24]. To better separate the sensitivity differences between *Ghsr^−/−^* and WT mice, we used CTA assays to assess the taste responsiveness to LA. Our results demonstrated that ghrelin-GHS-R signaling is involved in the lipid/fatty acid taste thresholds in mice, but future studies are still needed to explore additional tastants. While our CTA assay did not show changes in the LA thresholds of male mice, we did observe changes in the apparent LA thresholds of female mice. This is interesting in light of our data showing that loss of GHS-R in males leads to a reduction in body fat (*cf.* Figure 5B) but does not do so in females (Figure 5A). This suggests that there are significant sex differences in fatty acid taste and its metabolic regulation, a finding that has recently received additional empirical support [33]. Our results in *Ghsr^−/−^* mice, coupled with those of Cai et al. in ghrelin KO mice [12], may provide further insight into the role ghrelin plays in the taste system and whether it is through the GHS-R signaling pathway or through alternative mechanisms. Therefore, while it is clear from our data that ghrelin receptors are present in the peripheral taste system, whether the effects of loss of GHS-R in the present study are attributable to a direct action on the gustatory system or whether its regulatory effect is restricted to the descending central pathways remains unknown. Additional functional and mechanistic studies are needed to clarify the extent to which peripheral ghrelin directly targets the taste system and, more specifically, the pathways devoted to fat taste.

An important finding in this study is that GHS-R KO females demonstrated increased taste thresholds to LA, as assessed by CTA assays after the 6-week high-fat-diet feeding, while male mice showed no evidence of such an effect. While limited publications discuss the role of ghrelin in the taste system, research has shown sex-dependent effects of ghrelin on feeding behavior. Clegg et al. [34] reported increased food intake during peripheral injection of ghrelin in male rats, while no effects were seen in intact female rats. Additionally, females demonstrated reduced sensitivity to the orexigenic effects of centrally administered ghrelin. Furthermore, these sex-dependent effects of ghrelin were found to be estradiol mediated. Ovariectomized females displayed increased feeding in response to ghrelin; however, when given estradiol supplementation, the effects of ghrelin were again lost [34]. Previous studies also indicate that differences in taste preference exist between the two sexes. In a lickometer behavioral study using rats, ovariectomized female rats supplemented with estrogen responded to a lower LA + sucrose concentration than male counterparts [25]. LA can also increase the preference for lower monosodium glutamate (MSG) concentrations (40 mM) in male rats and higher MSG concentrations (100 mM) in female rats [35]. In one crowdsourcing human study, women and girls rated high concentrations of LA as more intense than men and boys [36]. Recently, it was shown that there are sex differences in fat taste detection and that estradiol acts as the key regulator in altering fatty acid taste responsiveness [33]. Females responded to lower concentrations of fatty acids than males, while loss of ovarian hormones reversed this effect by decreasing taste responsiveness to fat. Furthermore, fatty acid taste responsiveness varied significantly within the estrous cycle in females, where high levels of taste responsiveness coincided with high secretion of estradiol [33]. Of note, our CTA experiments illustrated similar fat taste thresholds between WT males and females, whereas significant differences were observed in previous studies [33,37]. A question of physiological interest is whether taste responsiveness is altered during the estrous cycle; therefore, it is possible that both the high-fat diet in our experiments and estrous cycle variation complicate apparent fat taste thresholds and contribute to these differences. Although the interplay between the effects of estradiol and ghrelin signaling in the taste system are uncertain, our data suggest that ghrelin may play a significant role in fatty acid detection in females and the interaction of both endocrine hormones may contribute to the observed sex differences. While the beginning of these effects may be seen in the slight decrease in the caloric intake of *Ghsr^−/−^* females, longer-term food intake studies in females need to be performed to better understand whether these effects lead to significant behavioral changes. Additionally, these changes in fat taste responsiveness may play a more significant role in preference when mice are presented different tastants simultaneously, but was not as apparent as only one choice (high-fat diet) was present.

Previous research shows that individuals with high fat sensitivity tend to consume less fat and gain less weight [38]. This suggests a negative correlation between fat taste threshold levels and food intake. However, we did not observe a similar pattern in *Ghsr^−/−^* females, as they showed decreased responsiveness to LA in the behavioral assay yet consumed slightly fewer calories than WT females. It is possible that much of the overall caloric reduction seen in these mice may be due to the central role of ghrelin/GHS-R. Central administration of ghrelin has been shown to increase caloric intake by acting on neuropeptide Y and agouti-related peptide [39]. This central role of ghrelin is well established and a potent driver of caloric intake. Central KO of GHS-R may be obfuscating the behavioral impact of ghrelin/GHS-R signaling that is present within the taste system. Further research is needed to better delineate the peripheral role of ghrelin/GHS-R in the taste system with central ghrelin/GHS-R signaling intact to better understand the importance of ghrelin signaling in the taste system.

## 5. Conclusions

In this report, we investigated ghrelin receptor expression patterns in taste cells and explored the change in LA taste thresholds and metabolic phenotypes in the presence and absence of growth hormone secretagogue receptor (GHS-R). Our results suggest that ghrelin-GHS-R signaling may have a direct action on the peripheral taste system, independent of descending central pathways. Additionally, ghrelin-GHS-R effects on the taste system appear to be sex specific, which may have important implications in differential weight regulation in men and women. Moreover, GHS-R and estrogen receptor (ERα) are highly co-expressed in a number of hypothalamic regions, indicating a dual role of GHSR and ERα in mediating metabolic signals [40]. ERα is also expressed in taste cells [33], and it is possible that estradiol signaling through ERα is convergent with GHS-R signaling in the taste system. These data help further elucidate the peripheral role of ghrelin in the taste system, likely linked to sex-dependent fatty acid taste pathways. Future studies exploring the mechanism by which ghrelin alters fat signaling in the taste system and its differential effects among the sexes will provide valuable insights into and understanding of the fundamentals of how endocrine factors affect taste perception and drive caloric intake.

## Figures and Tables

**Table 1:** Relative proportion of type II (PLCß2-positive) and type III (GAD67-positive) taste cells expressing GHS-R.

	**PLCß2-GFP, *n***	**GHS-R(+), *n***	**Co-Expressing, *n* (%)**
Circumvallate	101	97	69 (71.1)
Fungiform	12	8	8 (100)
	**GAD67-GFP, *n***	**GHS-R(+), *n***	**Co-Expressing, *n* (%)**
Circumvallate	114	103	3 (2.9)
Fungiform	9	24	1 (4.2)

## Data Availability

All relevant data are within the manuscript.
